# The screening of compounds regulating PD‐L1 transcriptional activity in a cell functional high‐throughput manner

**DOI:** 10.1002/cam4.5744

**Published:** 2023-03-25

**Authors:** Lanxin Zhang, Hexin Li, Jingchao Liu, Gaoyuan Sun, Xiaokun Tang, Siyuan Xu, Lili Zhang, Wei Zhang, Bin Ai

**Affiliations:** ^1^ Clinical Biobank, Beijing Hospital, National Center of Gerontology, National Health Commission Institute of Geriatric Medicine, Chinese Academy of Medical Sciences Beijing China; ^2^ Department of Urology, Beijing Hospital, National Center of Gerontology Institute of Geriatric Medicine, Chinese Academy of Medical Sciences Beijing China; ^3^ Department of Pathology, Beijing Hospital, National Center of Gerontology, National Health Commission Institute of Geriatric Medicine, Chinese Academy of Medical Sciences Beijing China; ^4^ Department of Medical Oncology, Beijing Hospital, National Center of Gerontology, National Health Commission Institute of Geriatric Medicine, Chinese Academy of Medical Sciences Beijing China

**Keywords:** cancer, high‐throughput screen, PD‐L1, vorinostat

## Abstract

Immune checkpoints are protein molecules expressed on the immune cell membrane, which regulate the immune system to kill tumor cells. As an essential immune checkpoint, overexpressed PD‐1 on tumor cells could inhibit T‐cell activation after being bonded to PD‐1. Due to this inhibitory effect, T‐cell proliferation and cytokine secretion are suppressed, leading to immune escape of tumor cells. Here, we established a high‐throughput method based on cell function screening technology to screen drugs regulating PD‐L1 expression in tumor cells at the transcriptional level. After two screening rounds, 12 compounds that enhanced PD‐L1 transcription while seven weakened were sorted out among 1018 FDA‐approved drugs. Finally, a tumor cell line was used to verify the upregulation of endogenous PD‐L1 expression for a drug named “vorinostat,” a histone deacetylation inhibitor, after the two rounds of optional selection. Therefore, our research provides another perspective for using “vorinostat” in treating tumors and offers a convenient method to detect the transcriptional expression of other intracellular proteins besides PD‐L1.

## Graphical abstract

The screening process of small molecular chemical drugs regulating transcriptional PD‐L1 via cell‐based high‐throughput screening system.

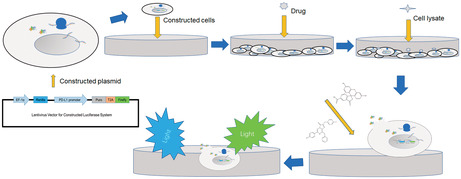

## 1 INTRODUCTION

Immune checkpoints are suppressive regulatory molecules in the immune system, essential for maintaining peripheral tolerance, preventing autoimmune reactions, and minimizing tissue damage by controlling the timing and intensity of immune responses.^1^, ^2^, ^3^ The expression of immune checkpoint molecules in immune cells inhibits the function of immune cells so that the body cannot produce an effective anti‐tumor immune response, eventually allowing the tumor to escape the immune system.^4^, ^5^

PD‐L1 belongs to the type I transmembrane protein with a size of 40 kDa, consisting of 290 amino acids constructed by IgV‐ and IgC‐like extracellular domains, a hydrophobic transmembrane domain, and a cytoplasmic tail.^4^, ^6^, ^7^ Constitutively, a low PD‐L1 expression could be found on antigen‐presenting cells (APCs), corneal, resting lymphocytes, and various nonhematopoietic cells that contribute to tissue homeostasis.^8^, ^9^ Also, as an essential immune checkpoint, PD‐L1, could be induced by inflammatory cytokines, including interferon (IFN), tumor necrosis factor‐α (TNF‐α), and vascular endothelial growth factor, as a suppressive signal on both endothelial/epithelial and hematopoietic cells.^10^, ^11^, ^12^ Additionally, PD‐L1 is overexpressed during malignancy for reasons of oncogenic driver events, such as epidermal growth factor receptor mutations, phosphatase‐tensin homolog (PTEN) mutations, and nucleophosmin (NPM)/anaplastic lymphoma kinase gene fusion, usually indicating a poor prognosis of tumor progression.^13^, ^14^, ^15^ PD‐L1 overexpressed on tumor cells can bind to PD‐1 on the activated T‐cell membrane, promoting tyrosine phosphorylation in the immune‐receptor tyrosine‐based switch motif (ITSM) domain of PD‐1, leading to the dephosphorylation of downstream protein kinases, Syk and PI3K, which then inhibit the activation of downstream AKT, ERK, and other pathways.^16^, ^17^ Eventually, the transcription and translation of genes and cytokines required for T‐cell activation are inhibited, and T‐cell activity is negatively regulated.^7^, ^18^

Small molecular chemical drugs significantly influence disease treatment. Although their development has recently reduced slightly, 38 small‐molecule chemical medicines approved in 2020 account for nearly 72% of the total number of new drugs approved.^19^, ^20^ Small molecular chemical drugs have the advantages of higher oral bioavailability, better pharmacokinetic characteristics, lower delivery difficulty, and lower production cost, which are conducive to their development.^21^, ^22^, ^23^ Reanalyzing the FDA‐approved small molecular chemical drugs could identify the new indications of those drugs, thereby shortening the R&D cycle and reducing the R&D cost, discovering ways to relieve drugs' side effects, or improving drug efficiency.^24^, ^25^ Therefore, we established a cell reporter system to screen a pool of FDA‐approved medicines that regulate PD‐L1 transcription based on a high‐throughput manner.

## 2 MATERIALS AND METHODS

### 2.1 High‐throughput screening of compounds regulating PD‐L1 expression at the transcriptional level

An A375 cell line (human A375 melanoma cell line which was purchased from ATCC) was used for high‐throughput screening to obtain compounds regulating the PD‐L1 promoter. A375 cells were seeded in 6‐cm dishes for 24 h to reach a 50%–60% confluence, and then the medium was refreshed for a better transfection environment. Lipofectamine LTX (Thermo Fisher Scientific Inc., Waltham, MA, USA) was used to transfect the prepared cells with a quantitative indicator of renilla luciferase‐expressing pWSLV 14‐renilla‐PDL1‐promotor‐firefly plasmid, according to instructions. After 24 h, cells were enzymatically disaggregated and reseeded into 96‐well plates at a density of 15,000 cells per well. Then, the medium was replaced with fetal bovine serum (FBS)‐free Dulbecco's Modified Eagle Medium (DMEM) after cells adhered to the wall. After that, 1018 FDA‐approved small molecular compounds were separately added at a final concentration of 10 μM and then the incubation was carried out. Forty‐eight hours later, the medium containing compounds was discarded, and the cells were washed with phosphate‐buffered saline (PBS) thrice. Cells with drug‐dissolving property were used as the control. Passive lysis buffer (PLB; Promega, Sydney, Australia) was used to lyse those treated cells for 20 min, and 20‐μL cell lysates were used to test the luciferase activity using a microplate reader (Synergy H1M, Thermo Scientific, Waltham, MA, USA), according to the instructions of the dual‐luciferase reporter gene system (Promega, Sydney, Australia).

### 2.2 Establishment of stabilized dual‐luciferase reporter cell lines for screening compounds that regulate PD‐L1 transcriptional expression

An A375 cell line with low PD‐L1 expression was used to establish stabilized cell lines for screening compounds that regulate PD‐L1 transcriptional expression. A PD‐L1 promoter was cloned from MDA‐MB‐231 cell lines and ligated to a lentivirus vector for constructing a PD‐L1 promoter‐overexpressed cell line, namely, A375‐PD‐L1 promotor cell line.

HEK‐293 T cells (human embryonic kidney 293 T cell line which was purchased from ATCC) were used for packaging lentivirus. Briefly, HEK‐293 T cells were plated in 10‐cm dishes for 24 h followed by transfecting a puromycin resistance screening indicator of Pac‐expressing and dual‐luciferase reporting lentivirus vector pWSLV 14‐renilla‐PDL1‐promotor‐luciferase and two package plasmids using Lipofectamine® 3000 reagent (Invitrogen, Carlsbad, CA, USA), according to its manufacturer's instructions. After 72 h, the medium containing the packaged virus was collected and centrifuged at 1500 rpm for 10 min. Sediments containing cellular fragments at the bottom of the tube were abandoned, while the culture supernatant was frozen below −80°C.

Wild‐type A375 cells were seeded into 10‐cm dishes, and the next day, the medium was refreshed following a gentle addition of drops of collected lentivirus. Seventy‐two hour later, a specific lethal concentration of puromycin to A375 was added to kill the uninfected A375 cells. After 10 days of puromycin resistance screening, the A375‐PD‐L1‐promotor cell line was established.

### 2.3 Dual‐luciferase reporter gene assay

The above established dual‐luciferase reporter system evaluated the activity of FDA‐approved small molecular compounds that regulate exogenous PD‐L1. IFN‐γ served as a positive control. Briefly, the A375‐PD‐L1 promotor cells were plated in 48‐well plates, and 24 h later, the medium was replaced with FBS‐free DMEM. The drug at final concentration gradients of 0, 0.1, 0.2, 0.5, 1, and 1.5 μM was added and separately incubated with those cells for 48 h. PLB was also used to dissociate those cells according to its manufacturer's instructions. Then, the cells were transiently centrifuged, and 20 μL was transferred to a 96‐well enzyme‐linked immunosorbent assay (ELISA) plate with three duplicates, then added to a microplate reader loaded with firefly luciferase reaction and renilla luciferase reaction working fluids. Detection was completed within 30 min.

### 2.4 Cellular toxicity test

Cell Counting Kit‐8 (CCK‐8, Dojindo Laboratories, Kumamoto, Japan) was used to test the cytotoxicity of screened small molecules to cells. A375 cells were seeded in 96‐well plates, then the medium was refreshed with FBS‐free DMEM as cells in the exponential growth phase. After 24 h, “vorinostat” (Selleck Chemicals LLC, Houston, TX, USA) at different final concentration gradients of 0, 0.025, 0.05, 0.125, 0.25, 0.5, 1, 2, 4, 8, 10, and 20 μM were added and incubated with those cells, respectively, for 48 h. Then, a final concentration of 10% (V/V) CCK8 was added to those wells and incubated at 37°C for 1.5 h. A microplate reader was used to monitor the absorbance of wells under 490 nm.

### 2.5 Reverse transcription quantificational polymerase chain reaction

Reverse transcription quantificational polymerase chain reaction (RT‐qPCR) was used to assess the transcription of endogenous PD‐L1 in A375 cells. Cells were seeded in 24‐well plates and managed as abovementioned. “Vorinostat” concentration used during assay were 0, 50, 100, 200, 400, and 800 nM and that for IFN‐γ were 0, 0.01, 0.05, 0.1, 0.2, and 0.4 μg/mL. Then, cells were washed thrice with PBS, and TRIzol (Invitrogen, Carlsbad, CA, USA) was added for cell lysis and RNA extraction. Trichloromethane (Tongguang, Beijing, China) was mixed with TRIzol, then the mixture was vortexed for 15 s and allowed to stand at room temperature (RT) for 3 min. The mixture was then centrifuged at 4°C and 14,000 rpm for 15 min. The supernatant was transferred into new tubes and mixed with isopropyl alcohol (Tongguang, Beijing, China) at RT for 10 min. Then, the tubes were centrifuged at 4°C and at a speed of 14,000 rpm for 10 min. The supernatant was discarded, and the residue was dried naturally after being washed by 75% alcohol three times. RNase‐free water was used to dissolve the obtained RNA precipitate. RNA reverse transcribing into cDNA was conducted according to FastQuant RT Kit instructions (With gDNase) (KR106) (Tiangen, Beijing, China) using T100™ Thermal Cycler (Bio‐Rad Laboratories, Inc., Hercules, CA, USA). Quantificational polymerase chain reaction was conducted following the procedures of FastFire qPCR PreMix (Probe) kit (Tiangen, Beijing, China) using q225 real‐time fluorescent quantitative PCR (Kubotechnology, Beijing, China). The reference gene used in this study was β‐actin, having “CATGTACGTTGCTATCCAGGC” as its forward primer sequence, and “CTCCTTAATGTCACGCACGAT” as its reverse primer sequence; the forward primer sequence for PD‐L1 was TGGCATTTGCTGAACGCATTT, and the reverse primer sequence was TGCAGCCAGGTCTAATTGTTTT.

### 2.6 Immunofluorescence analysis

Immunofluorescence (IF) was used to qualitatively measure the PD‐L1 protein expressed on A375 cells. As abovementioned, cells were seeded in 3.5‐mm laser confocal dishes and dealt with serum starvation. “Vorinostat” at a final concentration of 0.8 μM was added and incubated with those cells. After 48 h, cells were washed thrice with PBS, and every wash lasted for 5 min. Furthermore, 4% paraformaldehyde was applied for cell fixation. Then, rabbit‐anti‐goat primary PD‐L1 antibody (ab205921, Abcam, Cambridge, UK) at a ratio of 1:1000 was introduced to conjugate with PD‐L1 protein at 4°C overnight after the addition of 5% (M/V) Bovine serum albumin (BSA) blocking nonspecific antigenic buffer for 30 min at RT. Afterward, cells were washed with PBS to eliminate the uncombined primary antibody. Then, DyLight 488, a goat anti‐rabbit IgG secondary antibody (1:200 dilutions, A23220, Abbkine, CA, USA) was added to bind with primary antibody for 2 h at RT. Then, cells were washed with PBS thrice and stained with Hoechst 33342 (1:1000, Sigma‐Aldrich, St. Louis, USA) for 10 min at RT. Cells were then analyzed using an inverted fluorescence microscope (Eclipse‐Ti2, Nikon Inc., Japan) with excitation and emission wavelengths of 488 and 518 nm, respectively, after being washed with PBS.

### 2.7 Enzyme‐linked immunosorbent assay

The activity of small molecular compounds that upregulate endogenous PD‐L1 protein was evaluated by enzyme‐linked immunosorbent assay (ELISA) following the method outlined in the Human PD‐L1 Quantikine ELISA Kit (R&D Systems, Minneapolis, MN, USA) operation manual. Cells were plated in six‐well plates, and the medium was refreshed with FBS‐free DMEM after 24 h. “Vorinostat” (200 nM) was added incubated with those cells for 48 h. Then, cells were harvested and lysed to extract total protein. Within a 30 min period, using a microplate reader under 450‐nm wavelength, the optical density (OD) value was used to calculate the PD‐L1 protein expression (calibration wavelength 540 or 570 nm).

### 2.8 Western blot

Fresh cells were emptied of the culture‐medium and cleaned with pre‐cooled PBS, which was placed on the ice box at an inclined position. The remaining PBS in the petri dish was absorbed and added into the cell lysate. After standing on the ice for 20 min, cells were collected into the pre‐cooled EP tube with a gun. Supernatant was transferred to a new EP tube by ultrasound and centrifugation for 15 min. Protein concentrations were then determined using a BCA kit (Thermo Fisher Scientific Inc., Waltham, MA, USA). Finally, 5× Sample Buffer was added into the sample to be tested and boiled at 100°C for 5–7 min. Appropriate amount of protein was taken according to the protein concentration and then electrophoresis was performed. Electrophoretic bulk glue needs to be electrophoresed at 80 V for 30 min and separation glue at 100 V for 2 h. After electrophoresis was completed, proteins on gel electrophoresis were electrically transferred to the PVDF membrane by wet transfer method, and the membrane was transferred for 2 h at a voltage of 100 V on the ice. After completion of the membrane transfer, the PVDF membrane was closed with 5% skim milk powder prepared with PBST at room temperature for 1 h, and primary antibody (Thermo Fisher Scientific Inc., Waltham, MA, USA) was added for overnight incubation. TBST washed the membrane 5 times, 10 min each time, and then added secondary antibody for overnight incubation at 4°C. Finally, supersignal chemiluminescent substrate liquid A and liquid B were mixed at 1:1 and evenly smewed on the membrane, incubated for 4 min away from light, and exposed in dark room to show electrophoretic bands.

### 2.9 Flow cytometry

Flow cytometry (FCM) was used to quantitatively evaluate PD‐L1 protein expression on cell membrane. As abovementioned, cells were seeded in 12‐well plates and treated by serum starvation. “Vorinostat” with a final concentration of 200 nM was added and incubated with those cells for 48 h. Then, cells were harvested and washed thrice, 5 min each time, with a washing buffer (PBS containing 0.1% BSA). Soon afterward, cells were centrifuged at 1500 rpm for 5 min. The residue was suspended in APC anti‐human CD274 (B7‐H1, PD‐L1) antibody (5 μL per million cells in 100‐μL staining volume, Biolegend, San Diego, USA) for 30 min on ice followed by being washed thrice before flow cytometry (BD FACS Verse, Becton Biosciences, San Jose, CA, USA).

## 3 RESULTS

### 3.1 Construction of a luciferase reporter system for transcriptional PD‐L1 expression

**FIGURE 1:**
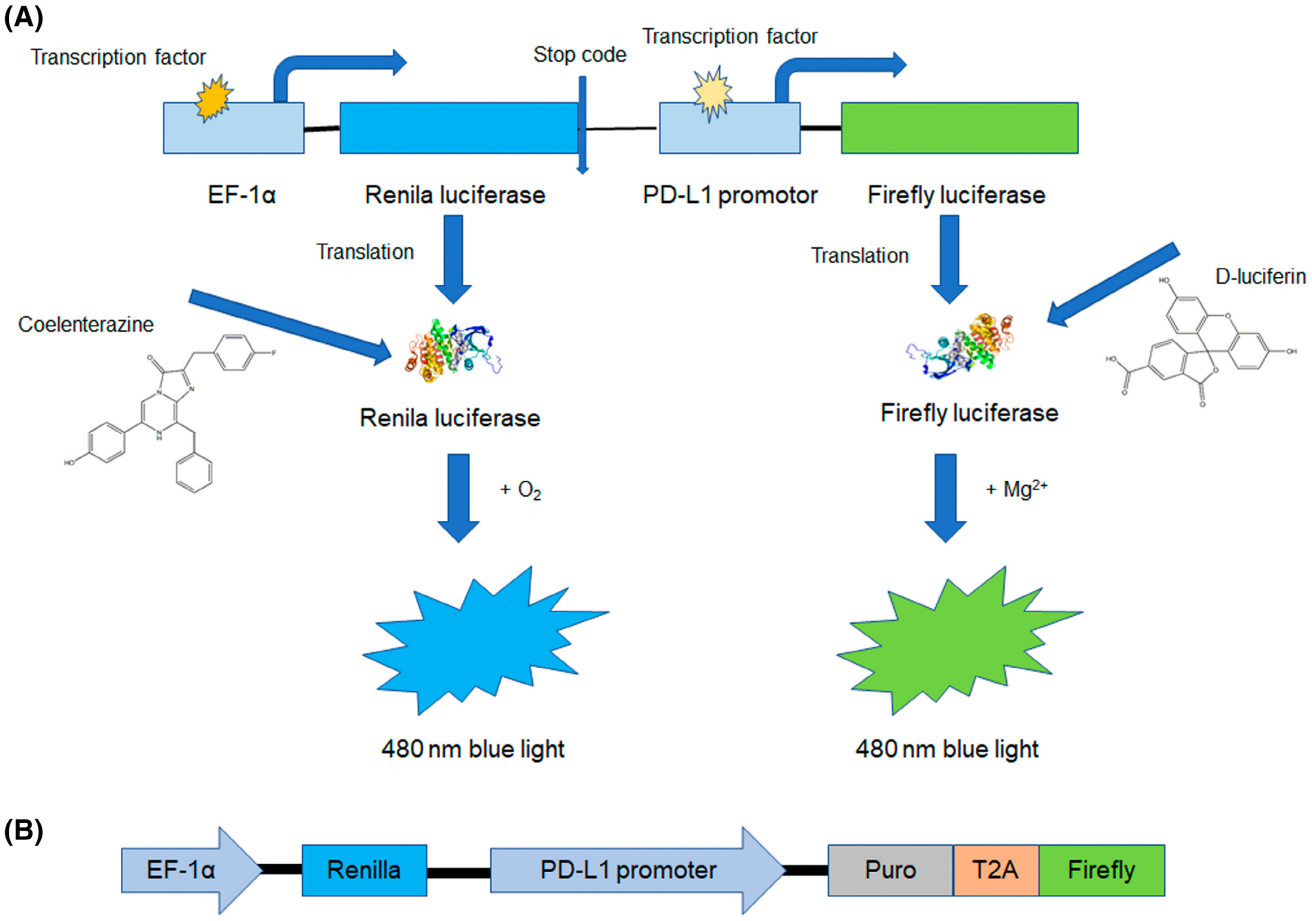
Schematic diagram of high‐throughput study for compounds regulating PD‐L1 transcription based on cell function screening system. The working mechanism of the established screening system was shown as (A), while the functional structure of the established plasmid was shown as (B).

Renilla and firefly luciferases were used to evaluate PD‐L1 transcriptional expression. As shown in Figure 1A, EF‐1α was used to initiate renilla luciferase activation, which uses an internal reference to normalize total effective cells. In contrast, the PD‐L1 promoter was used to activate the firefly luciferase, thus, representing the activity of small molecular compounds that act on the PD‐L1 promoter. A microplate reader was used to detect the light intensity of renilla and firefly luciferases, which was displayed as an optical density (OD) value. The evaluation index was measured in terms of fold change (FC) FC = [OD_firefly_ / OD_renilla_] _experimental group_ / [OD_firefly_ / OD_renilla_] _control group_. The constructed luciferase reporter system was shown in Figure 1B, and puro in a structure referred to pac gene for puromycin resistance selection.

### 3.2 Establishment of a stabilized A375‐PD‐L1‐promotor cell line

A stabilized A375‐PD‐L1‐promoter cell line was established using the constructed lentivirus vector was shown in Figure 1B to screen for FDA‐approved small molecular compounds, thereby regulating PD‐L1 transcriptional expression. Puromycin was used for resistance screening of successfully established cells. The effect of a series of puromycin concentrations on wild‐type A375 cells was tested, and a 50% lethal concentration of puromycin to wild‐type A375 was added to the above‐constructed A375‐PD‐L1 promoter cell and maintained for 10 days followed by the addition of 0.5 μg/mL puromycin during cell expansion.

### 3.3 Cell‐based high‐throughput screening

**FIGURE 2:**
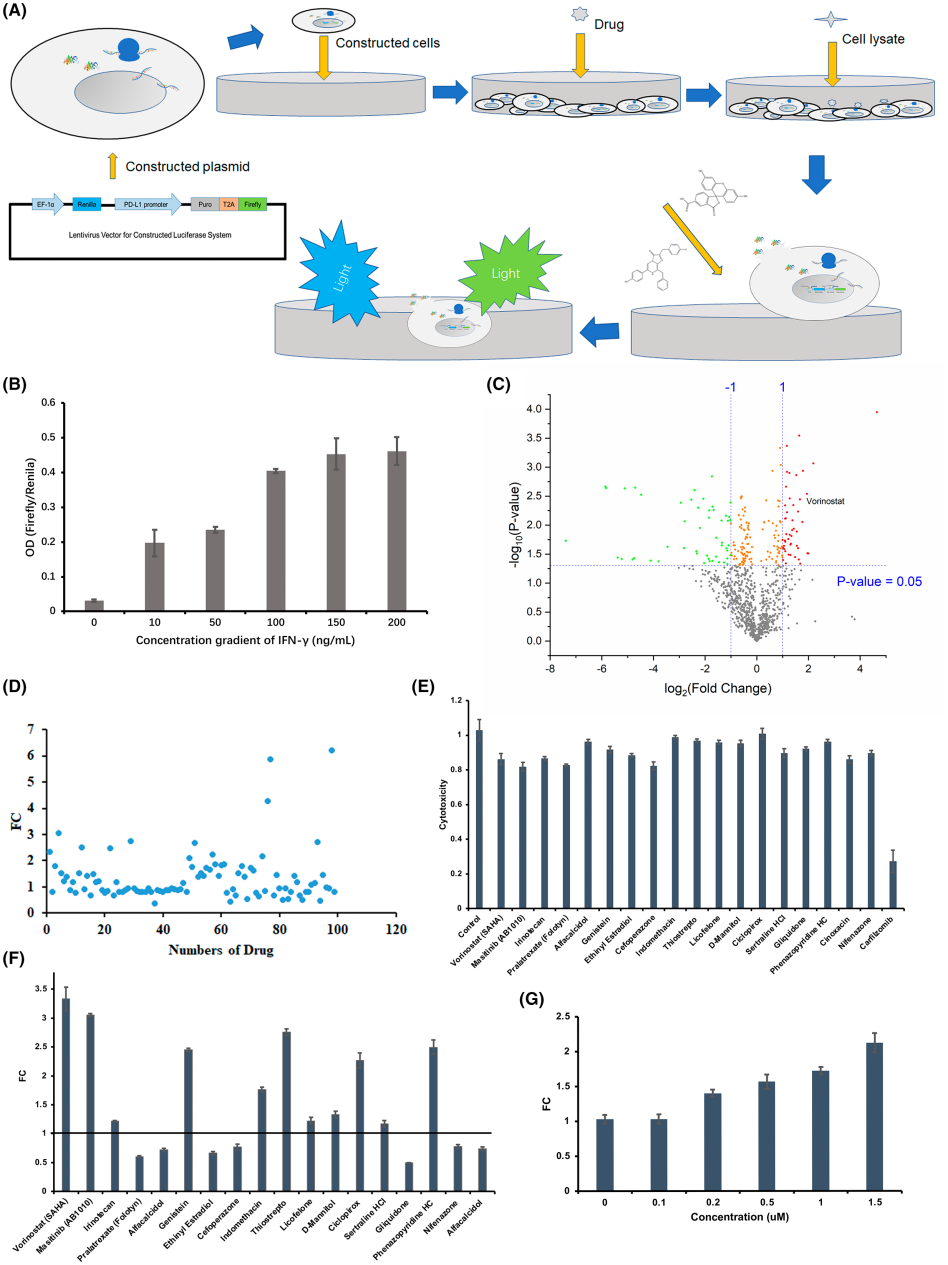
Screening of FDA‐approved small compounds in a high‐throughput manner. (A) Showed the screening process based on an established screening system. Validation of constructed screen system by IFN‐γ was shown as (B). The primary screening results of 1018 small molecular compounds were exhibited by volcano plot. The screening index of *p* < 0.05, FC>2 was set as the baseline for preliminary screening as shown in (C). (D) Revealed the results of the second round of screening specific to 98 compounds screened out in the primary screening. (E) Showed the cytotoxicity of 20 compounds picked up after a two‐round screen, while the results of the second round of screening specific to the 19 compounds except PR‐171 that were screened out during the primary screening was exhibited in (F). (G) Showed the representative compound screened after two rounds; vorinostat could positively act on exogenous PD‐L1 promoter in a dose‐dependent manner *N* = 3.

The primary screening workflow was shown in Figure 2A. The constructed plasmid was transfected into A375 cells to obtain transient expression of PD‐L1 promoter cell line and homodispersedly planted into 96‐well plates. One thousand and eighteen small molecular compounds were separately added and incubated with the above cells for 24 h, followed by PLB lysing cells to release renilla and firefly luciferases because they could not be secreted into the culture medium. Then, the coelenterazine substrate for renilla luciferase and D‐luciferin for firefly luciferase were added to react with luciferase.

To verify the validity of the constructed screening system, IFN‐γ has been reported to act on the PD‐L1 promoter to positively regulate PD‐L1 transcriptional expression and was incubated with the above cells. Consequently, FC values corresponding to the series gradient concentration of IFN‐γ are shown in Figure 2B. Along with the increment in IFN‐γ concentration, the FC value was also improved accordingly, thereby initiating the successful establishment of the constructed screen‐cell model. The results of the cell‐based high‐throughput screen are shown in Figure 2C. In the first screening round, 47 small molecular compounds upregulated and 51 compounds downregulated the exogenous transcriptional PD‐L1 expression based on the screening indices of FC > 2 and *p* < 0.05.

The second screening round specific to the above 98 compounds was performed as described above and the result is exhibited as shown in Figure 2D. After this step, 19 compounds that were similar to those generated in the first round were selected, of which 12 compounds upregulated transcriptional PD‐L1 expression. In comparison, eight compounds had an opposite effect on the PD‐L1 promoter. The cytotoxicity 6 of the above 20 compounds picked up after a two‐round screen is shown in Figure 2E. The selected 19 small molecular compounds nearly had no toxicity to the tested cells, except PR‐171, which induced cell apoptosis notably by inhibiting proteasome activity. The regulation ability of the 19 compounds except PR‐171 on the luciferase reporting PD‐L1 transcriptional expression system is shown in Figure 2F, there, a histone deacetylation inhibitor, “vorinostat,” showed the most vital ability among the abovementioned compounds to upregulate the exogenous transcriptional PD‐L1 expression.

### 3.4 Roles of “vorinostat” in endogenous PD‐L1 expression

**FIGURE 3:**
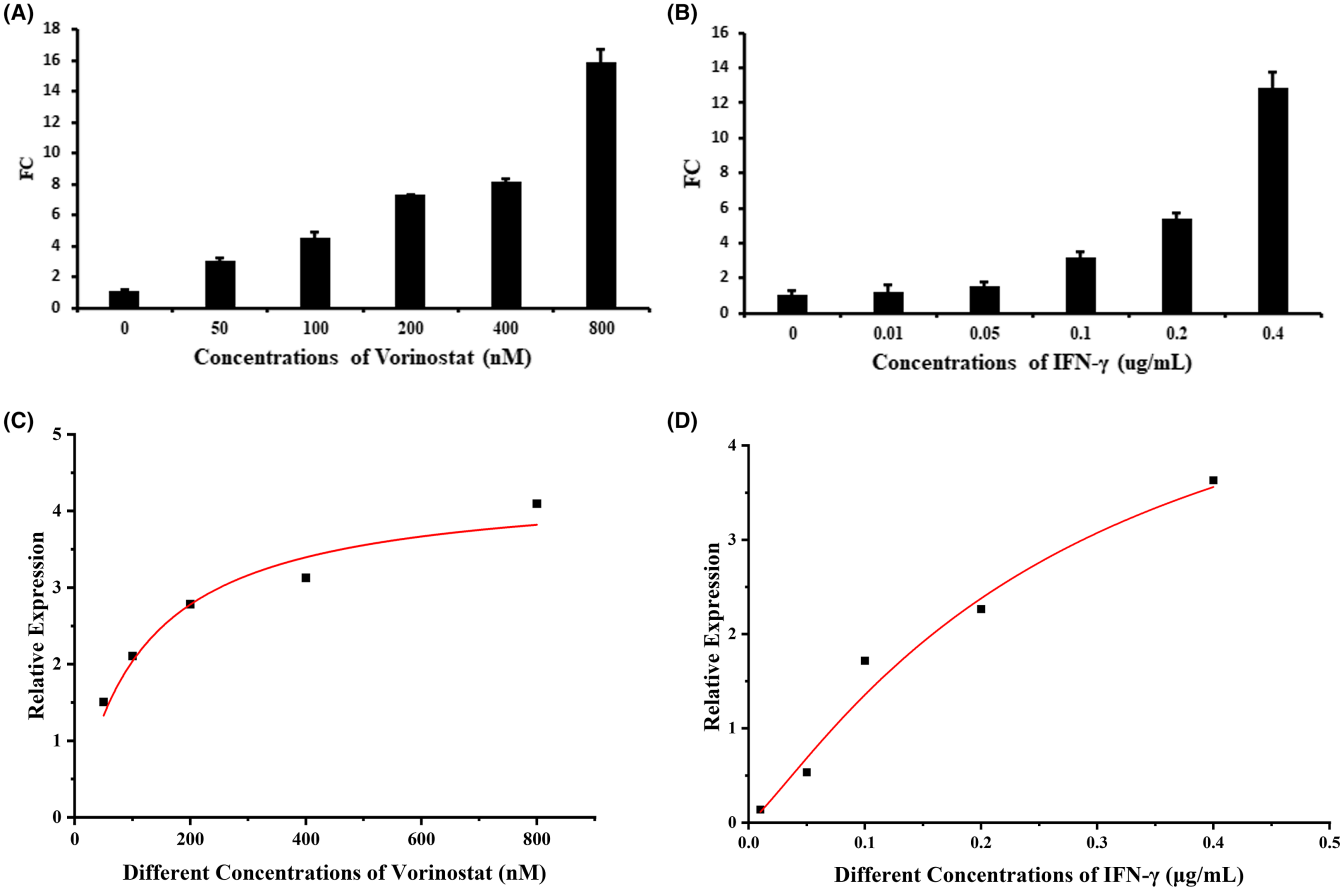
“Vorinostat” regulating endogenous PD‐L1 expression at the transcriptional level was evaluated by RT‐qPCR. The histogram of endogenous transcriptional PD‐L1 expression in A375 cells treated with different “vorinostat” or IFN‐γ concentration gradients are shown in (A, B) (*n* = 3), while the nonlinearly fitted vorinostat or IFN‐γ concentrations with a corresponding transcriptional PD‐L1 expression, as shown in (C, D), respectively.

Since “vorinostat” has been proven to positively act on exogenous PD‐L1 promotor, upregulating transcriptional expression of PD‐L1, wild‐type A375 was used to test the effect of “vorinostat” on endogenous PD‐L1, with IFN‐γ serving as a positive control. RT‐qPCR was used to evaluate roles of “vorinostat” in endogenous transcriptional PD‐L1 expression. The endogenous transcriptional PD‐L1 expression in A375 cells treated at different “vorinostat” or IFN‐γ concentration gradients are shown in Figure 3A,B, respectively. To get a detailed view of the results, the corresponding transcriptional PD‐L1 expression was nonlinearly fitted with “vorinostat” or IFN‐γ concentration (Figure 3C,D), respectively. The results show that “vorinostat” regulation of transcriptional PD‐L1expression in A375 cells was dose‐dependent, and the nonlinear curve fitting suggested that the regulation pattern of vorinostat was different from IFN‐γ.

**FIGURE 4:**
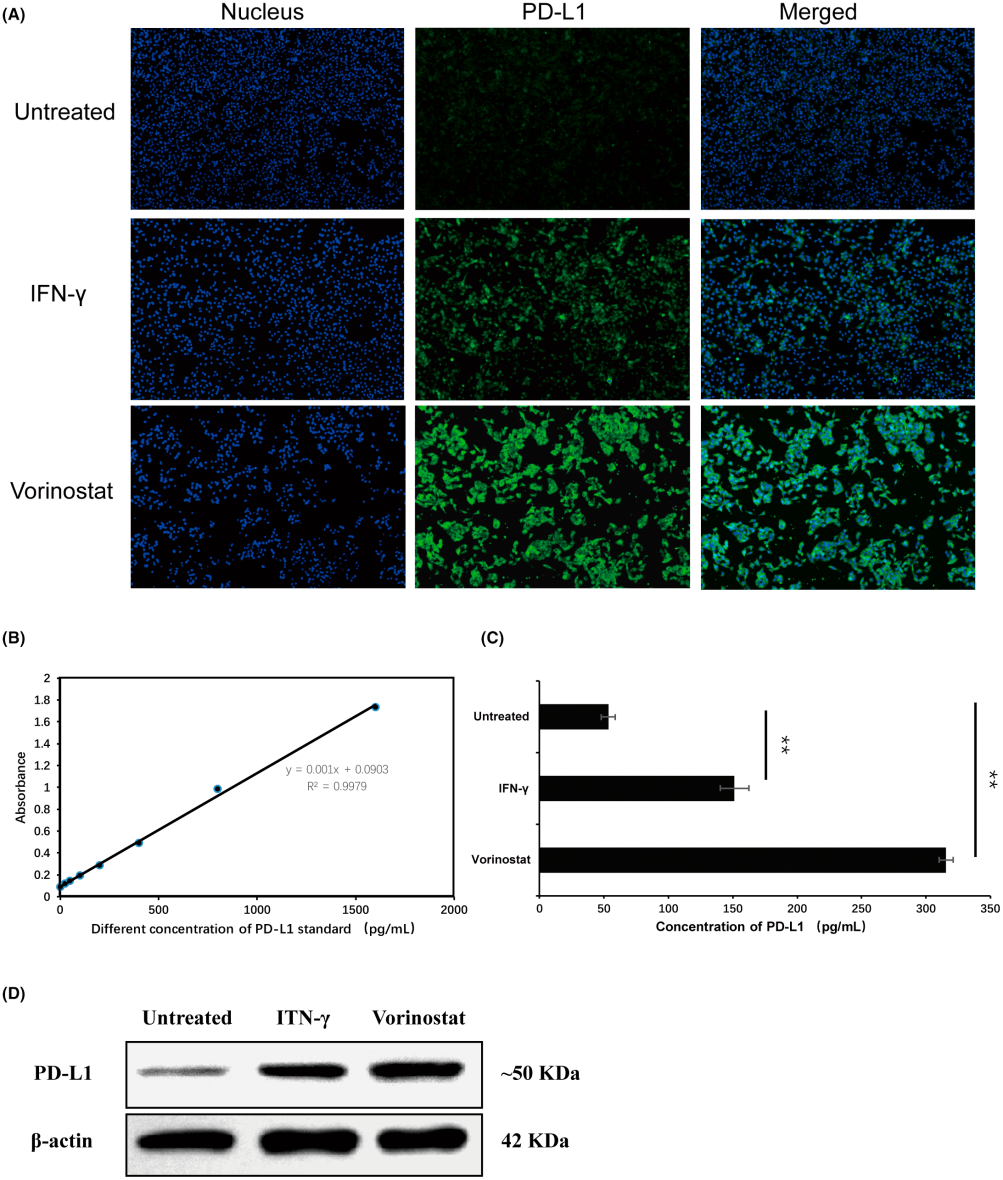
“Vorinostat” regulates endogenous PD‐L1 expression at the translational level. The qualitative endogenous PD‐L1 protein expression by IF, as shown in (A). ELISA was used to measure the total endogenous PD‐L1 expression at the translational level, as shown in (B, C) (*n* = 3). Asterisks (**) indicate *p* < 0.05 by *t*‐test while a scale bar in (A): 100 μm. Western blot was also used to measure total endogenous PD‐L1 expression as shown in (D).

Also, the endogenous protein expression of PD‐L1 regulated by “vorinostat” was investigated immediately after mRNA expression. As shown in Figure 4A, IF was used to qualitatively measure endogenous PD‐L1 protein expression with a dylight 488 conjugated secondary antibody‐stained PD‐L1 protein. The result showed that PD‐L1 endogenous expression in the “vorinostat”‐treated group was remarkably brighter than that in the control group. ELISA was used to measure the total expression of PD‐L1 protein expressed in A375. Before the measurement, the protein separately extracted from the different groups was quantitated using bicinchoninic acid. The standard curve for gradient concentrations of standard PD‐L1 protein and corresponding OD are shown in Figure 4B. The PD‐L1 protein concentration in each group was calculated by putting the measured OD of each group into the standard curve. As shown in Figure 4C, the expression level of total PD‐L1 protein in A375 cells treated with “vorinostat” was more than twice that of the control group.

**FIGURE 5:**
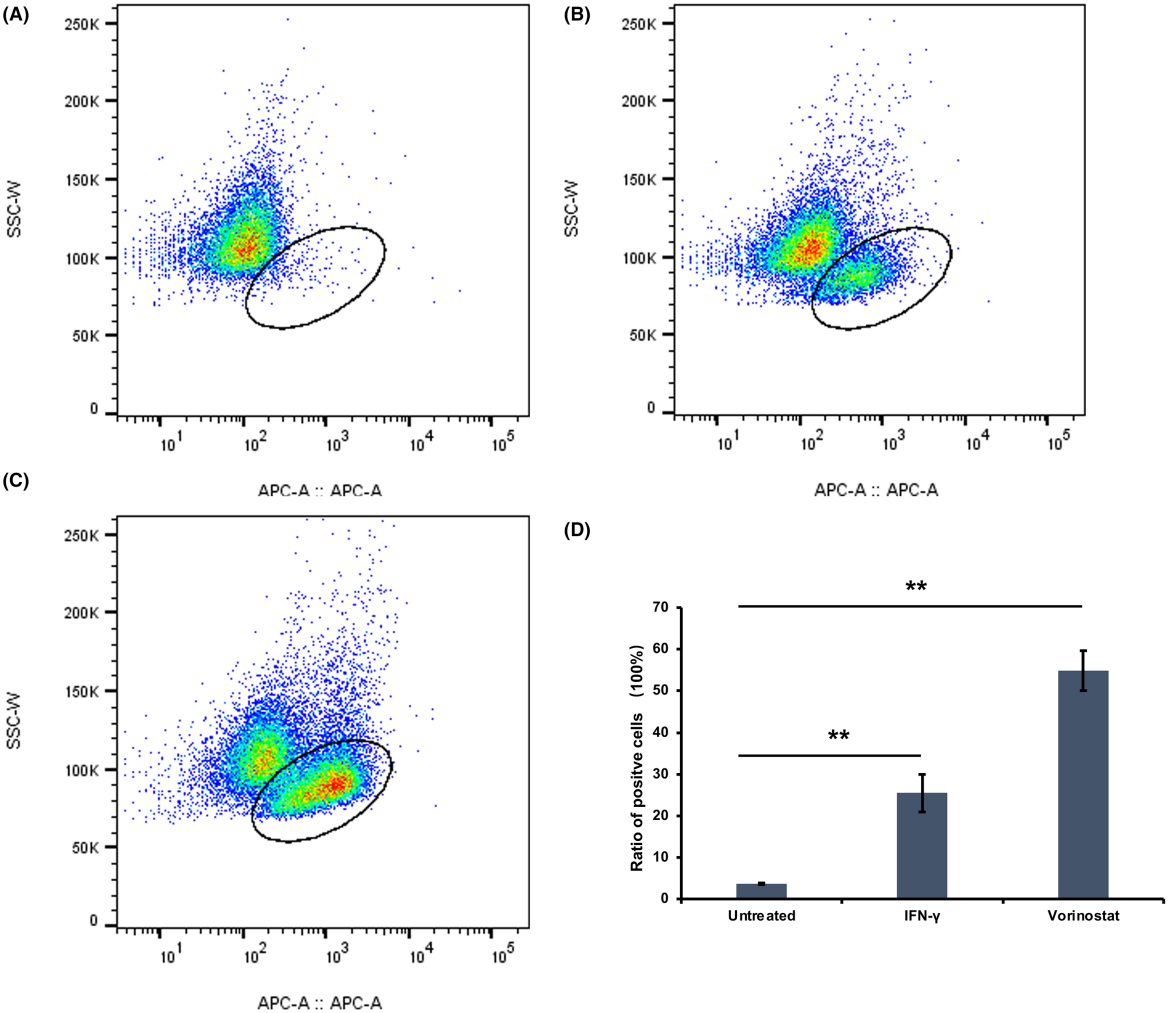
Membrane PD‐L1 protein could be upregulated by “vorinostat.” (A–C) Show the positive cell ratio detected by flow cytometry in untreated, IFN‐γ, and “vorinostat,” respectively. Simultaneously, the positive cell ratio in the different groups (*n* = 3) was compared as shown in (D). Ellipse regions indicate PD‐L1‐positive cells in (A–C), respectively. Asterisks (**) indicate *p <* 0.05 by *t*‐test.

PD‐L1 protein expressed on tumor cell membranes could bind to PD‐1 expressed on its membranes to mediate tumor cell immune escape; thereby, enabling the separate detection of the membrane PD‐L1 protein expression by flow cytometry. Furthermore, as shown in Figure 5, PD‐L1 protein expressed on the membrane increased about twice the control group after incubation with “vorinostat.”

## 4 DISCUSSION

PD‐L1 is a PD‐1 ligand.^26^ Its expression on tumor cell membrane could lead to a combination with PD‐1 expressed on T cells, and the resulting coupling signal could phosphorylate the immune receptor tyrosine–based switch motif (ITSM) domain, located in the cytoplasmic tail of the PD‐1 protein. Then, PD‐1 phosphorylation could dephosphorylate downstream protein kinase Syk and PI3K and further inhibit the activation of downstream AKT and ERK pathways.^27^, ^28^ Genes and cytokines needed to activate T cells were suppressed by PD‐1/PD‐L1 coupling, and negative regulation of tumor cells to T cells was ultimately achieved. Here, we established a cell‐based model to screen FDA‐approved small molecular compounds in a high‐throughput manner. After the two screening rounds, 12 compounds that upregulated the exogenous transcriptional expression of PD‐L1 and eight that downregulated PD‐L1 expression were selected. Finally, a histone deacetylase inhibitor, “vorinostat,” which showed the ability to upregulate exogenous PD‐L1 transcriptional expression, was tested in A375 to monitor its regulatory effect on endogenous PD‐L1.

At the end of this study, “vorinostat” enhanced PD‐L1 protein expression on the membrane of A375 cells instead of just improving PD‐L1 expression at the mRNA and intracellular protein levels. The overexpressed PD‐L1 on tumor cell membrane caused by “vorinostat” enhanced tumor cell resistance to T cells, mediated immune escape, and impaired the therapeutic effect of “vorinostat” to the tumor. The future clinical use of “vorinostat” to treat specific melanoma might increase the chances of tumor immune escape. Our study suggests that the therapy of combining “vorinostat” with PD‐1/PD‐L1 blockers as an adjuvant for treating melanoma was more effective than monotherapy involving just “vorinostat.”

In our cell‐based model, a dual‐luciferase reporter system was used to verify the effect of compounds to be screened. Renilla luciferase, initiated by the EF‐1α promoter, was used as a cell quantity indicator. In contrast, firefly luciferase was used to quantify the effect of PD‐L1 promoter compounds based on our constructed cell model. The OD value of firefly luciferase divided by renilla luciferase was used to evaluate the impact of compounds on the PD‐L1 promoter (OD firefly luciferase/OD renilla luciferase). FC was calculated by dividing the drug‐treated group by the untreated group ratios (FC = [test (OD firefly luciferase/OD renilla luciferase)/control (OD firefly luciferase/OD renilla luciferase)]). According to this theory, FC was exceeded by a mean for upregulation and reduced by a mean for downregulation. Our established cell‐based high‐throughput model can screen elements, such as compounds or siRNA regulating transcriptional PD‐L1 expression, and other protein regulation at the transcriptional level. However, this model has its limitation. Taking FC above 1, for example, means that OD values for firefly luciferase/renilla luciferase in the tested group, was bigger than that in the control group. There are two explanations for this event, firefly luciferase was activated by the PD‐L1 promoter (upregulation) and renilla luciferase was activated by the EF‐1 promotor (downregulation). Thus, implies that the test drug may affect the FC by positively regulating the PD‐L1 promoter or negatively regulating the EF‐1 promoter. Therefore, our established cell model was used only in the first few screening rounds to reduce the workload; thus, an endogenous experiment must be conducted to further verify the screening results.

In conclusion, we established a cell‐based model to screen an FDA‐approved drug pool in a high‐throughput manner. Using this cell model, we discovered that the regulatory expression of PD‐L1 functions at the transcriptional level for those “old” drugs. After the two screening rounds, 19 compounds were selected. Finally, the histone deacetylase inhibitor, vorinostat, was discovered to regulate endogenous PD‐L1 expression at the transcriptional and translational levels. This designed novel screening system targeting protein expression at transcriptional level would provide new means for compounds and siRNA screening. Conclusively, our research offers a strategy for protein‐related screening at the transcriptional level and suggests that the combination with PD‐1/PD‐L1 blockers would achieve better clinical efficacy than using just “vorinostat” for monotherapy.

## AUTHOR CONTRIBUTIONS

**Lanxin Zhang:** Conceptualization (lead); data curation (lead); formal analysis (lead); project administration (equal); supervision (lead); writing – original draft (lead); writing – review and editing (equal). **Li Hexin:** Conceptualization (lead); data curation (lead); formal analysis (equal); project administration (equal); writing – original draft (equal); writing – review and editing (equal). **Jingchao Liu:** Data curation (equal); formal analysis (equal); investigation (equal); project administration (equal); supervision (equal); writing – original draft (equal); writing – review and editing (equal). **Sun Gaoyuan:** Formal analysis (equal); project administration (equal); resources (equal); supervision (equal); validation (equal); writing – review and editing (equal). **Tang Xiaokun:** Formal analysis (equal); project administration (equal); supervision (equal); writing – original draft (equal); writing – review and editing (equal). **Xu Siyuan:** Conceptualization (equal); data curation (equal); writing – original draft (equal); writing – review and editing (equal). **Zhang Lili:** Conceptualization (equal); investigation (equal); supervision (equal); writing – review and editing (equal). **Zhang Wei:** Conceptualization (equal); funding acquisition (lead); investigation (equal); project administration (equal); supervision (lead); writing – original draft (equal); writing – review and editing (lead).

## CONFLICT OF INTEREST STATEMENT

None.

## Data Availability

Data sharing is not applicable to this article as no new data were created or analyzed in this study.
